# Dual targeting of conserved cell cycle and transcription programs in advanced colorectal cancer by fadraciclib

**DOI:** 10.1093/emph/eoaf021

**Published:** 2025-08-08

**Authors:** Mohammad Zokaasadi, Wylie K Watlington, Divya L Dayanidhi, John B Mantyh, Gabrielle Rupprecht, Shannon McCall, David G Blake, Jason A Somarelli, David S Hsu

**Affiliations:** Department of Medicine, Division of Medical Oncology, Duke University Medical Center, Durham, NC, USA; Center for Genomics and Computational Biology, Duke University, Durham, NC, USA; Department of Medicine, Division of Medical Oncology, Duke University Medical Center, Durham, NC, USA; Center for Genomics and Computational Biology, Duke University, Durham, NC, USA; Department of Medicine, Division of Medical Oncology, Duke University Medical Center, Durham, NC, USA; Center for Genomics and Computational Biology, Duke University, Durham, NC, USA; Department of Medicine, Division of Medical Oncology, Duke University Medical Center, Durham, NC, USA; Center for Genomics and Computational Biology, Duke University, Durham, NC, USA; Department of Medicine, Division of Medical Oncology, Duke University Medical Center, Durham, NC, USA; Center for Genomics and Computational Biology, Duke University, Durham, NC, USA; Department of Pathology, Duke University, Durham, NC, USA; Cyclacel Ltd, Dundee, UK; Department of Medicine, Division of Medical Oncology, Duke University Medical Center, Durham, NC, USA; Center for Genomics and Computational Biology, Duke University, Durham, NC, USA; Department of Medicine, Division of Medical Oncology, Duke University Medical Center, Durham, NC, USA; Center for Genomics and Computational Biology, Duke University, Durham, NC, USA

**Keywords:** patient-derived organoids, Fadraciclib, colorectal cancer, cyclin dependent kinases

## Abstract

**Background and objectives:**

Control of cell division is tightly regulated in eukaryotic cells, and dysfunction in cell cycle checkpoints is a key hallmark of malignant transformation that promotes a fitness advantage over non-cancer cells. One of the most critical mechanisms of cell cycle regulation is via the cyclin-dependent kinases (CDKs), which connect resource availability sensing and growth signaling with cell division and transcription elongation processes. Novel combination therapy approaches to co-target cell cycle and transcriptional CDKs may improve cancer-specific targeting of CDK dysfunction. In the current study, we assessed the effectiveness of fadraciclib, a new CDK2/9 inhibitor, for the treatment of advanced colorectal cancer (CRC).

**Methodology:**

A panel of eighteen CRC patient-derived organoids (PDOs) was used to assess the efficacy of fadraciclib. Efficacy was further validated in patient-derived xenografts (PDXs). CDK2/9 target inhibition, cell cycle arrest, and cell killing mechanisms were investigated using western blotting, flow cytometry, and immunofluorescence staining, respectively.

**Results:**

CRC PDOs exhibited greater sensitivity to fadraciclib compared to chemotherapy and palbociclib. This efficacy was validated *in vivo* using three matched PDXs, showing significant tumor growth inhibition with fadraciclib compared to vehicle (*P* < .05) and no serious adverse effects. Fadraciclib induced G2/M cell cycle arrest, leading to multipolar mitosis and anaphase catastrophe.

**Conclusions and implications:**

Our results using patient-derived models suggest that fadraciclib is a promising therapy for advanced CRC by inhibiting CDKs 2 and 9, which affects critical pathways in cell cycle regulation and transcription.

## INTRODUCTION

Cancer is a speciation event within the body in which a cell gains a fitness advantage over its non-cancer counterparts [1]. The cancer cell acts as a unicellular species within a dynamic ecological system (the host), with a population of genetically diverse individuals competing for space and resources [2]. To successfully compete, cancer cells must possess key phenotypes, known as cancer hallmarks. These hallmarks help to ensure survival of the cancer species, but they also represent potential vulnerabilities. Two of the major hallmarks of cancer are dysregulated cell division (proliferation) and genome instability. As the sole means of reproduction for the cancer cell, an elevated rate of cell division ensures the survival of the population by providing a fitness advantage over non-cancer cells. An increased proliferation rate is critical for providing a selective advantage, particularly during primary tumor growth, as exhibited by truncal alterations in cell cycle checkpoint molecules (TP53, CDKN2A) and drivers of cell division (e.g. KRAS, Myc, and PI3K signaling [3].

While a higher cell division rate would confer a selective advantage by enabling specific subclones to proliferate more rapidly and overtake a population, there are also fitness tradeoffs to increased rates of cell division, including a higher rate of DNA breaks or mutations and an increase in energy expenditure to complete cell divisions. The accumulation of DNA breaks can lead to chromosome instability and gain in chromosome number, which can lead to dysfunction in chromosomal segregation, anaphase catastrophe, and apoptosis [4]. Although the higher chromosome instability resulting from dysregulated proliferation can have a disadvantage for the *individual*, the increased chromosome instability can also confer a selective advantage at the *population level* by increasing genetic diversity within the population. Cancer cells can overcome this disadvantage at the individual cell level by upregulation of cyclin-dependent kinase 2 (CDK2). CDK2 is a key factor in mediating proper alignment and segregation of supernumerary chromosomes, thus enabling cells within the population to survive a level of DNA alteration that would otherwise induce cell death. The increase in CDK2 activity therefore increases the fitness of the individual by promoting its continued survival and reproduction.

The higher proliferation rate in cancer cells also requires other cellular systems to meet these demands, and there is increasing evidence of alterations in transcription and translation rates in cancer cells to maintain expression of genes necessary to sustain the increased proliferation rate of these cells [5, 6]. Among these processes, sustained transcription is a key feature of cancer in which cells upregulate and activate genes and proteins necessary to promote transcription elongation, including CDK7, CDK9, and CDK12/13 [7]. These transcriptional CDKs ensure continued production of transcripts necessary to sustain the increased proliferation potential, thereby leading to increased fitness for the cancer cell.

While this increase in cell division rate provides a fitness advantage for cancer cells over non-cancer cells, it also presents a vulnerability to agents that can selectively target differential cell division rates between cancer and non-cancer cells. Consistent with this, many early chemotherapeutics were discovered based on their ability to broadly target rapidly proliferating cells. These chemotherapies have been a mainstay of treatment for cancer; however, they also exhibit significant toxicity, as their non-specific targeting of cell division also leads to the death of dividing non-cancer cells (e.g. hair, gut, reproductive system, and bone marrow) [8]. To avoid some of these side effects, agents have been developed that more specifically target CDKs that are dysfunctionally upregulated in cancer to control rapid cell division while relying on functional redundancies in the CDK gene family in non-cancer cells to limit toxicity. Multiple CDK inhibitors are approved for the treatment of various cancers, including palbociclib, ribociclib, abemaciclib, and trilaciclib [9].

Despite these limited successes, however, many of the strategies that target a single dysregulated process, such as increased proliferation, have failed to show substantial benefit, as the diversity of the cancer cell population enables rapid evolution of resistance mechanisms [10] . An alternative strategy—borrowing from infectious disease strategies [11]—is to co-target a combination of phenotypes necessary for maintenance of increased fitness. Indeed, in our prior studies we provided evidence that this could be achieved by targeting CDK2 and CDK9 as a potential combination therapy for colorectal cancer (CRC) [12]. CDK2/9 inhibition has also been shown to induce anti-proliferative effects in preclinical models of lung cancer [13] and acute myeloid leukemia [14]. Co-targeting multiple vulnerabilities in these models led to cell cycle arrest, anaphase catastrophe from mis-segregation of supernumerary chromosomes, and subsequent apoptosis [13, 14].

In the present study, we interrogate the potential of dual CDK2/9 inhibition as a novel anti-proliferative strategy to treat CRC. Using a panel of 18 CRC patient-derived organoids (PDOs), we evaluate the efficacy of fadraciclib, a novel orally bioavailable dual CDK2/9 inhibitor. Patient-derived models of cancer (PDMC), such as patient-derived xenografts (PDXs) and PDOs are becoming standard preclinical models in precision medicine because of their improved capability over cell lines in recapitulating the tumor microenvironment, intratumoral heterogeneity, and response to treatment at the individual patient level [15, 16]. Using a series of PDXs, we further show that fadraciclib significantly impairs tumor growth rate *in vivo*. Mechanistically, we observe inhibition of Rb and RNA polymerase II phosphorylation, increased rates of anaphase catastrophe, and G2/M cell cycle arrest. Together, these results indicate that fadraciclib may be a promising novel therapy for the treatment of CRC that takes advantage of key phenotypes of cancer cells for therapeutic benefit.

## METHODOLOGY

### Development of patient-derived organoids, drug screening, viability assays and synergy assays

The platform and process for establishment of patient derived organoids has been previously described [17]. Subsequent to organoid establishment, CRC media was aspirated from the wells, washed using phosphate buffered saline (PBS) to detach organoids and Matrigel from the bottom of the wells, and centrifuged for 5 min at 750 × *g*. Following aspiration of supernatant, 1 ml TryplE Express enzyme (Gibco) was added to dissociate organoids to single cells and dissolve the Matrigel. After 5 min of incubation at 37°C, quenching media consisting of DMEM supplemented with 10% fetal bovine serum (FBS) and 100 U/ml Penicillin/Steptomycin was added, and cells were counted, mixed with Matrigel and plated at a density of 5 × 10^3^ cells per 5 μl dome in each well of a 96-well plate. The organoids were kept in CRC media (50 μl per well) after the domes were solidified and incubated overnight. Drugs were added the following day in a seven-point dose curve ranging from 6.4 nM to 100 μM. Cell viability was quantified after 72 h using the Cell-Titer Glo (CTG) luminescent Cell Viability Assay kit (Promega). IC_50_ values were calculated using non-linear curve fit with the log (inhibitor) vs. response (three parameters) in GraphPad Prism. Area under the curve (AUC) values were also calculated for all conditions and AUC values of 500 and 0 were considered as no response and no growth under any conditions respectively. Live cell imaging was done using Alexa Fluor® 647 conjugated rabbit monoclonal epithelial cell adhesion molecule (EpCAM) antibody (Abcam#ab237396) at 1:1000 concentration. ImageXpress® Pico (Molecular Devices, USA) was used for imaging, and the plates were read in Varioskan Lux plate reader (Thermo Fisher Scientific).

For synergy studies, 5 μl domes were plated, and drug combinations were added the next day in 2×2 dose matrix of serial dilutions from 160 nM to 100 μM using a fixed dilution factor of 5 for CVT-313 (CDK2 inhibitor, SelleckChem) and 20 nM to 100 μM using a fixed dilution factor of 3 for LDC000067 (CDK9 inhibitor, SelleckChem). Subsequently, cell viability was analyzed after 72 h using CTG. Synergy was calculated using SynergyFinder2.0 [18]. We used the zero interaction potency (ZIP) model to assess synergy. ZIP is a novel model to capture synergy which compares the potency of each individual drug to the combination of the drugs and generates a score system in which scores >10 show synergy, scores <-10 showing antagonism, and scores in between are indicative of additive effect [19].

### Extraction of whole cell lysates and western blotting

A total of 5 × 10^4^ cells were plated in 50 μl domes in each well of a 24-well plate. After 5 days, organoids were treated with vehicle dimethyl sulfoxide (DMSO), 1× or 3× the average IC_50_ value of fadraciclib. Organoids were harvested after 24 h of exposure to drug. Matrigel was depolymerized using cell recovery solution (Corning), and organoids were lysed using radioimmunoprecipitation assay lysis buffer supplemented with phosphatase and protease inhibitors to extract total protein. Total protein quantities were measured with BCA protein assay (Bradford). A total of 80 μg of lysate was separated via electrophoresis on 4%–15% polyacrylamide gels (Biorad) and transferred to polyvinylidene difluoride membranes. Membranes then were blocked for 1 hour in 5% non-fat dry milk solution in tris buffered saline with 0.1% tween and then incubated with primary antibodies (anti Rb, Abcam#ab181616, anti phospho-Rb (Ser807/811), Cell Signaling#9308, anti RNA Pol II, Active Motif#39097, and anti phospho-RNA Pol II (Ser2), invitrogen#MA5-32637) overnight in 4°C. The following day membranes were washed, and appropriate secondary antibodies were added. Blots were imaged using a Licor Odyssey imaging system.

### Cell cycle analysis

A total of 5 × 10^4^ cells were plated in each well of a 12-well plate (five 20 μl domes per well, 1 × 10^4^ cells per dome). After 5 days, when approximately 60% confluent, organoids were treated with 1/2× or 1× IC_50_ doses of fadraciclib. After 72 h of drug exposure, organoids were detached and dissociated to single cells, fixed in 80% ice cold ethanol, re-suspended in PBS, and treated with RNase A, 20 μg/ml, for 90 min at 37°C. Finally, cells were stained with propidium iodide, 50 μg/ml for 15 min in the dark. Flow cytometry was performed in the Duke Cancer Institute flow cytometry facility at Duke University which is supported by the NCI Cancer Center support grant award number P30CA014236.

### Multipolar mitosis assays

A total of 1 × 10^6^ cells dissociated from organoids were suspended in 10 ml RPMI media supplemented with 10% FBS and 100 U/ml Penicillin/Streptomycin were plated in a tissue culture treated dish to generate matched 2D cell lines. After adherence of cells, a total of 3 × 10^4^ cells were plated in each well of a 24-well plate and treated with either DMSO or fadraciclib. After 24 h of incubation with drug, cells were fixed for 15 min in 4% paraformaldehyde and permeabilized for 30 min in 0.2% Triton-X 100 in PBS. After blocking in 5% bovine serum albumin in PBS for 1 h, primary antibodies [α-tubulin (DM1A), mouse monoclonal, Cell Signaling #3873; and γ-tubulin, rabbit monoclonal, Abcam #ab179503] were used to label target proteins at concentrations recommended by the manufacturer. The cells were incubated overnight at 4°C. The following day, primary antibodies were aspirated and washed with PBS. Secondary antibodies (anti-Mouse IgG H&L, Alexa Fluor® 488, Cell Signaling #4088, and anti-Rabbit IgG H&L, Alexa Fluor® 647, Abcam#ab150079) and DNA counterstain were incubated for 1 hour at room temperature in the dark, after which cells were washed and left in PBS. Imaging and quantification were done with ImageXpress® Pico (Molecular Devices, USA) and the number of events was calculated in 5% of the surface area of each well.

### Development of patient-derived xenograft model and *in vivo* drug sensitivity studies

Following establishment of PDXs as described earlier [20] they were harvested, mechanically digested and 5 × 10^6^ cells were re-injected in 100 µL of PBS. When tumors reached 250 mm^3^, mice were randomized to different treatment groups. A minimum of three mice were used in each treatment condition per PDX. We maintained a 1:1 male-to-female ratio in our mice cohort for each PDX. Vehicle consisted of 100 μl ultrapure distilled water (Invitrogen). Fadraciclib was administered at 25 mg/kg in 100 μl ultrapure distilled water (Invitrogen), BID via oral gavage. Tumor sizes were measured using a digital caliper. Tumor sizes and animal weights were measured thrice weekly. Treatments were administered for 5 days a week and continued for 2 weeks.

### Statistical analysis

Statistical analyses were performed in GraphPad Prism (La Jolla, CA, USA). Differences between groups were analyzed with t-tests for two groups and analysis of variance (ANOVA) with Tukey’s *post hoc* analysis for more than two groups. A chi-square test was used to analyze quantified immunofluorescent images of multipolar mitosis events between vehicle and treatment groups. *P*-values <.05 were considered statistically reliable. Fluorescent intensity analysis was done using library “imager” in R software v4.4.1 for Windows.

## RESULTS

### Establishment of patient-derived models of CRC

**Figure 1:**
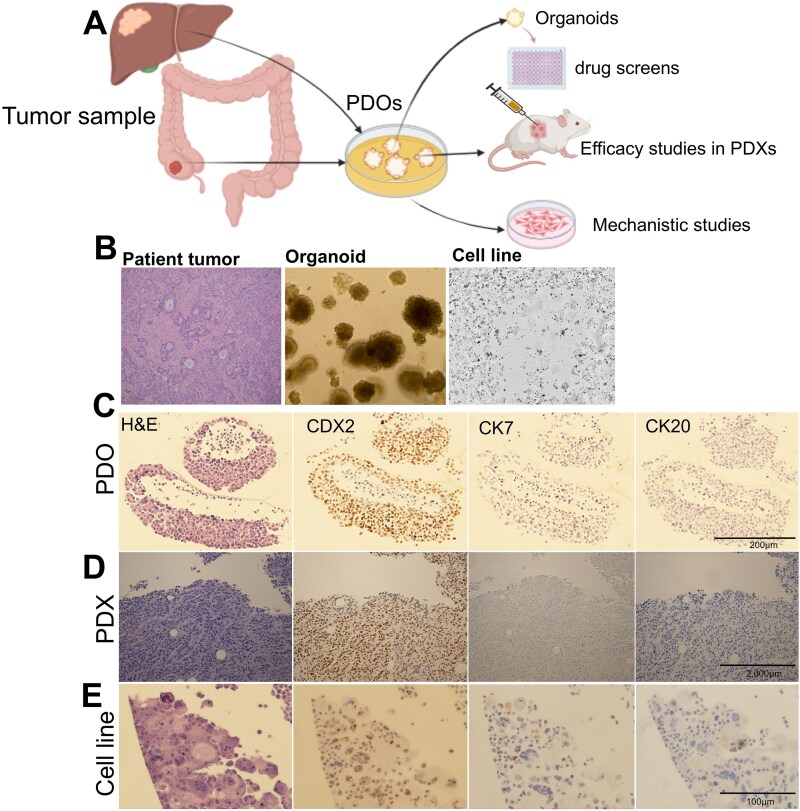
Precision oncology platform using PDMC. (A) Schema of our precision oncology platform using matched PDO, PDX and early passage cell lines from patient sample (either primary or metastatic) to perform *in vitro* drug screenings followed by *in vivo* validation and mechanistic studies. (B) Representation of a matched patient tumor, PDO and early passage cell line. H&E and IHC demonstrated that PDO 18 (C), PDX 18 (D), and matched cell line (E) are pathologically similar to the patient tumor from which they were derived.

In the current study, we used a series of PDMC to explore the efficacy in targeting the cell proliferation hallmark as a vulnerability to treat CRC. To do this, we established a series of CRC PDMC, including matched PDOs, PDXs, and early-passage cell lines for our study platform (Fig. 1A). The platform uses PDOs to evaluate drug efficacy, PDXs to validate *in vivo* therapeutic efficacy, and PDOs and early-passage matched cell lines for mechanistic studies (Fig. 1A). A total of 18 PDOs were included in the study. Histological analysis by immunohistochemistry (IHC) confirmed the presence of CRC in the PDMC (Fig. 1B–E).

### Fadraciclib is an effective anti-tumor agent in CRC PDOs

**Figure 2:**
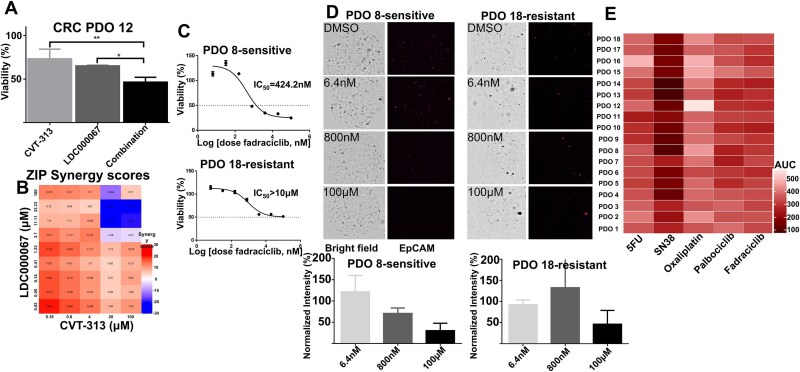
*In vitro* anti-cancer efficacy of dual CDK2/9 inhibition. (A) Viability of PDO12 after 72 h of exposure to CVT-313, LDC000067 (100 μM) and combination revealed that combinatorial therapy is more effective than single agent CVT-313 (ANOVA, *P* < .01) or LDC000067 (ANOVA, *P* < .05). (B) ZIP synergy analysis confirms synergy in a 2 × 2 dose matrix of CDK2 and CDK9 inhibitors. (C) Fadraciclib IC_50_ curves for the most sensitive CRC PDO (PDO 8) and most resistant CRC PDO (PDO 18), respectively. (D) Bright field and EpCAM fluorescent images, after 72 h of incubation with different doses of fadraciclib depicting the sensitivity and resistance of the PDOs. Quantification of signal intensity confirms relative sensitivity and resistance in PDO 8 and PDO 18 lines, respectively. (E) Heatmap showing AUC for all lines included in the study. AUC values of 500 and 0 are representative of no response and no growth under any conditions, respectively.

Our previous study identified CDK2/9 inhibition as a potentially promising therapeutic target for CRC [12]. In order to assess the synergy of dual CDK2/9 inhibition in CRC PDOs, we tested the efficacy of CVT-313 (CDK2 inhibitor) and LDC000067 (CDK9 inhibitor) alone and in combination in CRC PDO 12. Figure 2 shows that dual inhibition of CDK2 and CDK9 resulted in synergistic cell growth inhibition in dose ranges <4 μM (Fig. 2A), with ZIP synergy scores >10, indicating synergistic interaction between these agents (Fig. 2B). Based on these results, and consistent with our previous observations [12], we sought to test fadraciclib, a clinical-stage, orally bioavailable CDK2/9 inhibitor, across a broader panel of eighteen CRC PDOs.

IC_50_ curves were first generated across our panel of CRC PDO (Supplemental Fig. SS1) with IC_50_ values ranging from 300 nM to >10 μM (Supplemental Table SS1). Quantification of cell viability by CTG pinpointed both drug-sensitive and -resistant PDOs (Fig. 2C). We defined resistant PDOs as organoids that do not achieve 50% cell killing for the highest drug concentration. For resistant lines in which IC50 values could not be estimated, the AUC was calculated for these lines and all lines included in the study (Fig. 2E). To further verify the relative sensitivity of these PDOs, we implemented a live cell imaging platform with EpCAM to longitudinally track cell growth inhibition in response to CDK2/9 inhibition. Consistent with our results using CTG, real-time imaging of PDO growth using EpCAM showed similar growth inhibition upon treatment with fadraciclib (Fig. 2D). A total of 14/18 (78%) CRC PDOs were sensitive to fadraciclib with IC_50_ values <1.0 μM and mean IC_50_ of 569 ± 182 nM. The 4/18 resistant PDOs had IC_50_>10 μM. We conducted a correlation analysis between different SOC and fadraciclib. The only significant correlations between fadraciclib and other agents were *R*^2^ = 0.28 (*P* < .05), and *R*^2^ = 0.33 (*P* < .05) for SN38 and palbociclib, respectively.

### Fadraciclib is efficacious in multiple CRC PDX models *in vivo*

To validate the *in vivo* efficacy of fadraciclib, we next treated four CRC PDX with fadraciclib. First, tolerability of SCID-beige mice to fadraciclib was tested by the administration of fadraciclib via oral gavage at doses of 25 mg/kg daily, 25 mg/kg BID and 50 mg/kg daily to 5 mice in each dose. Two deaths (40%) occurred in the 50 mg/kg group, and the mice in this group exhibited significant weight loss (21.52% ± 9.47%) requiring euthanasia before the end of the study in accordance with our IACUC protocols. In contrast, mean weight loss was 1.78% ± 3.88% in the 25 mg/kg BID group and 5.00% ± 1.27% in the 25 mg/kg daily group (Supplemental Fig. SS2A). Based on these observations, we selected 25 mg/kg BID to evaluate efficacy.

**Figure 3:**
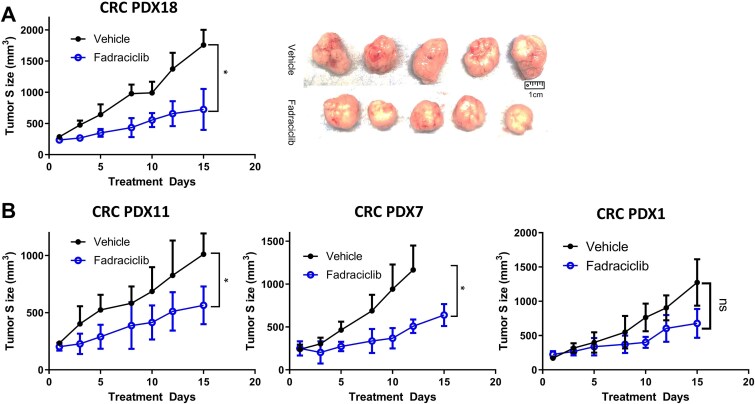
*In vivo* efficacy of fadraciclib. (A) CRC PDX 18 treatment results with either vehicle or fadraciclib showed a significant tumor growth inhibition between treatment and vehicle groups after 2 weeks of treatment. (t-test, *P* < .05). (B) Significant tumor growth inhibition is also detected in CRC PDX 7 (left panel) and 11 (middle panel) (t-test, *P* < .05) and a trend toward significant tumor growth inhibition is seen in CRC PDX1 (t-test, *P* = .14).

Treatment of CRC PDX18 from a metastatic CRC patient with fadraciclib led to significant tumor growth inhibition compared to the vehicle (Fig. 3A). Treatment of three additional CRC PDX (1, 7, and 11) generated from primary CRC also exhibited significant tumor growth inhibition in two of three PDXs (CRC PDX 7 and 11; Fig. 3B, *P* < .05) with a trend toward decreased tumor growth in PDX1 (Fig. 3B; *P* = .14). In PDX7 the tumors in the vehicle group were ulcerated; thus, euthanized for humane endpoints before the end of the designated 2-week treatment time. Importantly, no additional toxicities, such as weight loss, diarrhea, limb weakness, anxiety/restlessness, or hair loss, were observed in the mice treated with fadraciclib. Mean weight loss for the animals during the study ranged between 7.63% and 12.50% of the baseline weight (Supplemental Fig. SS2B-E). Collectively, these *in vitro* and *in vivo* results indicate that dual CDK2 and 9 inhibition may be a promising and tolerable therapy for patients with primary or metastatic CRC.

### Fadraciclib inhibits Rb and RNA pol II phosphorylation, resulting in anaphase catastrophe and cell cycle arrest

**Figure 4:**
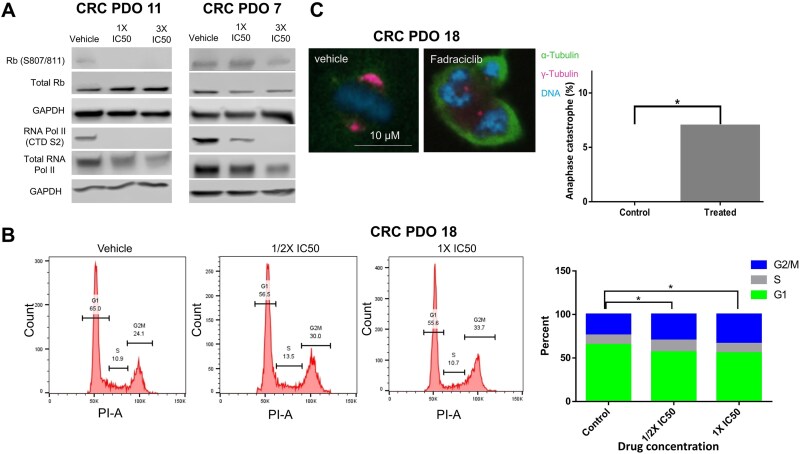
Mechanism of action for fadraciclib. (A) Western blot analysis of CDK 2 and CDK 9 substrate proteins after exposure of CRC PDO 11 and 7 to fadraciclib revealed reduced phosphorylation of serine 2 residue on RNA polymerase II CTD and of serine 807/811 residues on Rb protein. (B) Induction of cell cycle arrest after 3 days of treatment with different doses of fadraciclib, (ANOVA, *P* < .05). (C) Anaphase catastrophe in CRC PDO 18 after treatment with fadraciclib compared to normal bipolar mitosis. Quantification of events showed a significant difference between treated and control groups (chi-square, *P* < .05).

To determine mechanisms by which fadraciclib may trigger cell death we performed western blots for phosphorylation of serine 807/811 on Rb (CDK2 target) and serine 2 on the carboxy terminal domain (CTD) of RNA pol II (CDK9 target). We observed downregulation of both phosphorylation sites on CDK2 and CDK9 substrates after 24 h of treatment with fadraciclib (Fig. 4A). Fadraciclib treatment also resulted in downregulation of total RNA pol II (Fig. 4A), which has been demonstrated previously; however, the downregulation in phosphorylated forms was more robust [14].

Inhibition of CDK2, which is known to regulate the progression from G1 through S phase to G2, induces potent cell cycle arrest [21–23], while CDK9 is involved in transcription control by regulating RNA polymerase II. Therefore, to confirm target specificity of CDK2/9 inhibition, we first analyzed cell cycle progression in four CRC PDO lines (1, 7, 11, and 18) treated with ½ X and 1X IC_50_ dose of either vehicle or fadraciclib. Cell cycle analysis on four PDO lines (1, 7, 11, and 18) revealed a significant increase in G2/M arrest in all lines treated with fadraciclib (Fig. 4B; *P* < .05; Supplemental Fig. SS3A–C).

Fadraciclib has been shown to induce anaphase catastrophe via formation of multipolar mitosis [24]. To determine if fadraciclib has a similar mechanism of action in CRC, we analyzed supernumerary centrosomes by immunofluorescence from three early passage cell lines using γ-tubulin as a centrosomal marker and found a significant increase in supernumerary centrosomes (Fig. 4C; Supplemental Fig. S3D–E; *P* < .05).

## DISCUSSION

Unchecked proliferation is one of the major phenotypes by which specific cancer cells outcompete non-cancer and cancer cells for space and resources. In CRC, this increase in proliferative capacity is often driven by activation of Ras (BRAF, KRAS, etc.) [25–28], PI3K signaling [29,30], or loss of cell cycle checkpoints, such as APC or p53 [31–34], as well as p16, p21, and multiple CDKs [35]. While this increased proliferation renders cancer cells with a selective advantage over non-cancer cells, it also provides a potential therapeutic window to selectively target cancer cells for eradication through administration of CDK inhibitors that target multiple cellular processes important for cell proliferation, in this case cell division and transcriptional elongation. Herein we combined the advantages of PDMC to evaluate the efficacy of targeting cell division and transcriptional elongation with the novel, orally bioavailable CDK2/9 inhibitor, fadraciclib, for the treatment of CRC. Fadraciclib acts by inducing cell cycle arrest [12] and anaphase catastrophe [13,36] through inhibition of CDK2 [24]. During the cell cycle, CDK2 inactivates Rb via phosphorylation, allowing cells to proceed through the cell cycle [14]. Inhibition of CDK2 promotes cell cycle arrest by preventing phosphorylation and subsequent inactivation of Rb. CDK2 inhibition also prevents phosphorylation of the centrosomal protein, CP110 [37], by CDK2, which results in accumulation of multiple centrosomes [37]. Collectively, inhibition of CDK2 can cause Rb-dependent cell cycle arrest and promote centrosomal deregulation and subsequent induction of anaphase catastrophe. Since aneuploidy is common in many human cancers [38], this mechanism provides a potential therapeutic window for targeting cancer and sparing non-cancer cells. Consistent with this, previous studies have shown that fadraciclib has a more anti-proliferative effect in lung cancer cell lines as compared to normal epithelial cells [36] and greater potency in acute myeloid leukemia [39] as compared to hematopoietic cells.

Along with CDK2 inhibition, fadraciclib also acts on CDK9 to halt the transcriptional machinery, which leads to a reduction in labile proteins, such as the anti-apoptotic protein MCL-1, in leukemia and solid tumor cell lines *in vitro* [29], and both in PDX models of lung cancer [36] and in patients receiving fadraciclib by a single infusion [40]. In this study, oral fadraciclib dosed at 25 mg/kg BID showed improved tolerability over daily 50 mg/kg dosing in mice. A phase 1/2 study (NCT04983810) is currently evaluating oral BID dosing of fadraciclib in subjects with solid tumors or lymphoma. Dosing at 25 mg/kg BID was efficacious in PDX models and could benefit from improved exposure; fadraciclib dosed by a single 4 h infusion in a phase 1 safety trial (NCT02552953) of patients with advanced solid tumors showed a half-life of 1.64 to 3.9 h [40]. Further optimization of preclinical dosing to extend the half-life of this agent may provide additional benefit.

## CONCLUSION

Overall, fadraciclib seems to be a potentially efficacious agent for the treatment of CRC by co-targeting dual upregulation of cell division (CDK2) and transcriptional (CDK9) checkpoints. These checkpoints, when aberrantly activated are necessary to promote a fitness advantage for cancer cells over non-cancer cells and other cancer cell subclones that lack this cell cycle and transcriptional upregulation. Future studies should be aimed at precision-guided clinical evaluation of CDK2/9 inhibition in CRC and other solid tumors.

## Supplementary Material

Supp_Table_eoaf021

Supplemental_Figure_1_eoaf021

Supplemental_Figure_2_eoaf021

Supplemental_Figure_3_eoaf021

Supplemental_Caption_eoaf021

## Data Availability

The data sets of the current study are available from the corresponding author on reasonable request.
