# Supplementary material for: Dual targeting of conserved cell cycle and transcription programs in advanced colorectal cancer by fadraciclib
Source: Evol Med Public Health. 2025 Aug 8;13(1):281–90. doi: 10.1093/emph/eoaf021 (PMC12507023; doi:10.1093/emph/eoaf021)
Supplement: Supp_Table_eoaf021 [file supp_table_eoaf021.docx]

| **Estimated IC_50_ (μM)** | | | | | |
| --- | --- | --- | --- | --- | --- |
| **Sample ID** | **5-FU** | **SN-38** | **Oxaliplatin** | **Palbociclib** | **Fadraciclib** |
| CRC PDO 1 | >100 | 0.077 | 8.81 | 38.266 | >10 |
| CRC PDO 2 | >100 | 0.027 | 100 | 11.581 | >10 |
| CRC PDO 3 | >100 | 0.151 | 9.368 | 27.72 | >10 |
| CRC PDO 4 | >100 | 0.018 | >100 | 14.562 | 0.351 |
| CRC PDO 5 | >100 | 0.074 | >100 | 7.492 | 0.389 |
| CRC PDO 6 | 2.932 | 0.166 | 7.895 | 20.085 | 0.393 |
| CRC PDO 7 | 3.737 | 0.086 | 5.638 | 6.038 | 0.399 |
| CRC PDO 8 | 10.085 | 0.035 | 100 | 3.722 | 0.424 |
| CRC PDO 9 | 1.16 | 0.057 | 22.322 | 4.723 | 0.446 |
| CRC PDO 10 | 1.231 | 0.032 | >100 | 10.534 | 0.506 |
| CRC PDO 11 | 0.535 | 0.033 | 11.61 | 6.259 | 0.575 |
| CRC PDO 12 | 7.874 | 0.027 | >100 | 9.535 | 0.622 |
| CRC PDO 13 | 6.085 | 0.028 | 17.147 | 4.092 | 0.624 |
| CRC PDO 14 | 5.081 | 0.044 | 35.092 | 2.949 | 0.625 |
| CRC PDO 15 | 100 | 0.187 | >100 | 10.335 | 0.814 |
| CRC PDO 16 | 7.513 | 0.061 | 10.825 | 8.083 | 0.868 |
| CRC PDO 17 | 4.313 | 0.117 | 14.565 | 5.957 | 0.926 |
| CRC PDO 18 | >100 | 0.021 | 20.765 | 30.087 | >10 |
