# Supplementary figures and images for: Dual targeting of conserved cell cycle and transcription programs in advanced colorectal cancer by fadraciclib
Source: Evol Med Public Health. 2025 Aug 8;13(1):281–90. doi: 10.1093/emph/eoaf021 (PMC12507023; doi:10.1093/emph/eoaf021)

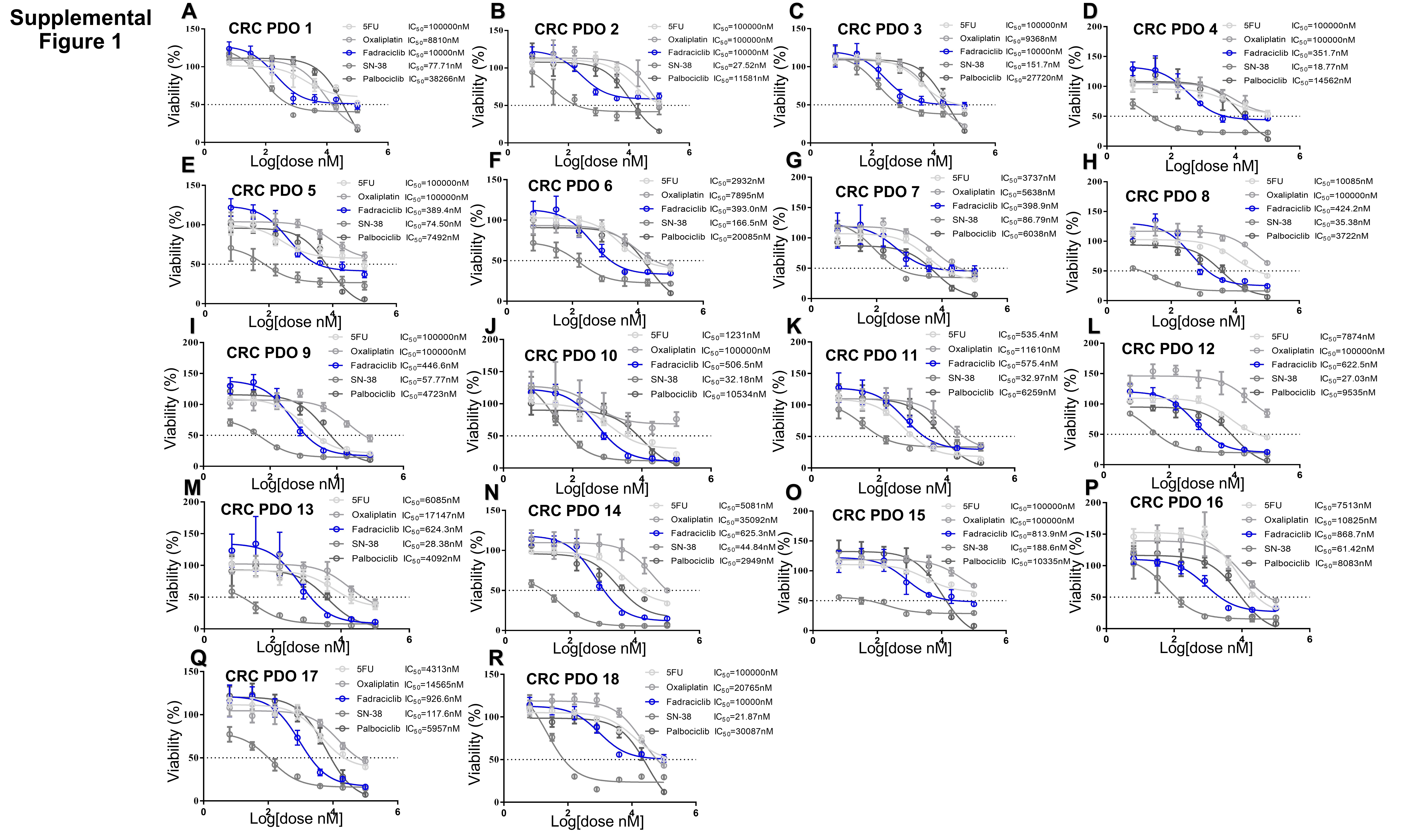

Supplement: Supplemental_Figure_1_eoaf021 [file supplemental_figure_1_eoaf021.jpeg]

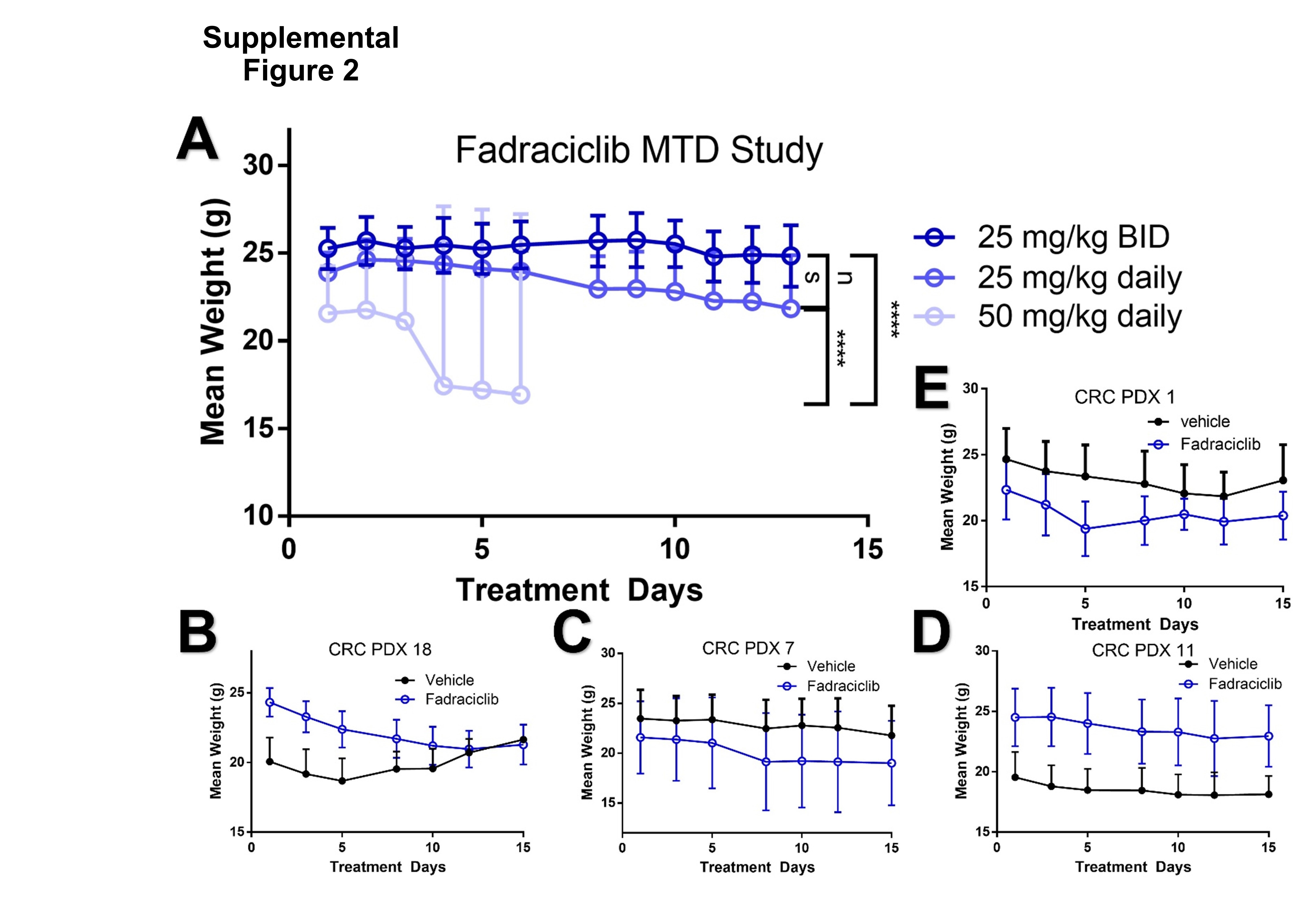

Supplement: Supplemental_Figure_2_eoaf021 [file supplemental_figure_2_eoaf021.jpeg]

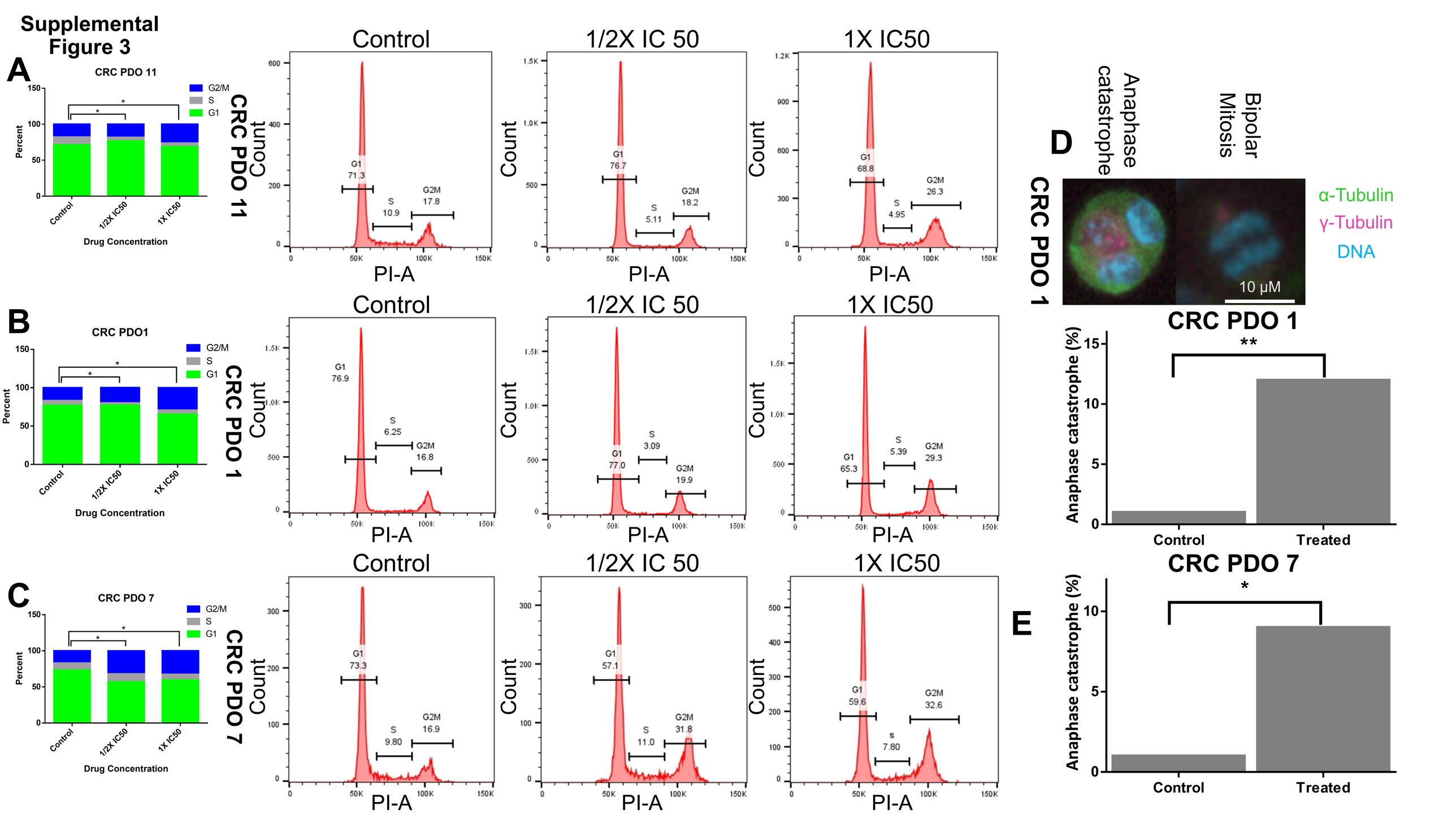

Supplement: Supplemental_Figure_3_eoaf021 [file supplemental_figure_3_eoaf021.jpeg]
