# Supplementary material for: Dual targeting of conserved cell cycle and transcription programs in advanced colorectal cancer by fadraciclib
Source: Evol Med Public Health. 2025 Aug 8;13(1):281–90. doi: 10.1093/emph/eoaf021 (PMC12507023; doi:10.1093/emph/eoaf021)
Supplement: Supplemental_Caption_eoaf021 [file supplemental_caption_eoaf021.docx]

**Supplemental Table.** IC_50_ values for all the lines included in the study.

**Supplemental Figure 1.** IC_50_ curves for all PDO lines used in the study showing the similar pattern of lower values for fadraciclib compared to SOC. (p<0.05)

**Supplemental Figure 2.** A. Fadraciclib tolerability was tested in SCID-beige mice in order to select the drug dosage for in vivo efficacy experiments. The highest tolerated dose tested was 25 mg/kg BID. Body weight loss in the 50 mg/kg group was significantly higher compared to 25 mg/kg daily and BID groups, ANOVA test, p<0.001. There was also a 40% mortality rate among the 50 mg/kg group. No deaths were observed in other groups. B to E, mean body weight among treated mice for PDX lines 1, 7, 18 and 11 at 25 mg/kg BID fadraciclib.

**Supplemental Figure 3.** A-C. Induction of cell cycle arrest in CRC PDO 11, 1 and 7 lines. After 3 days of incubation with different concentrations of fadraciclib all three models showed an increased proportion of cells in G2/M (p<0.05) D and E. representative IF images in addition to quantification of anaphase catastrophe events between treated and untreated groups of PDO-derived cell lines (7 and 1) showed a significant difference between treatment and control groups (p<0.05).
